# Threading the Needle: Navigating Novel Immunotherapeutics in Pancreatic Ductal Adenocarcinoma

**DOI:** 10.3390/cancers17050715

**Published:** 2025-02-20

**Authors:** Tarik Demir, Carolyn Moloney, Devalingam Mahalingam

**Affiliations:** Developmental Therapeutics, Robert H. Lurie Comprehensive Cancer Center, Northwestern University Feinberg School of Medicine, Chicago, IL 60611, USA; tarik.demir@nm.org (T.D.); carolyn.moloney@nm.org (C.M.)

**Keywords:** biomarkers, immunology, immunotherapy, pancreas adenocarcinoma, resistance mechanisms

## Abstract

Pancreatic ductal adenocarcinoma (PDAC) is a lethal malignancy with a poor prognosis. Currently, chemotherapy is the only option for most patients with advanced-stage PDAC. Further, conventional immunotherapies and targeted therapies improve survival outcomes only in rare PDAC patient subgroups. To date, combinatory immunotherapeutic strategies to overcome the immune-hostile PDAC tumor microenvironment (TME) have resulted in limited efficacy in clinical studies. However, efforts are ongoing to develop new treatment strategies for patients with PDAC with the evolving knowledge of the TME, molecular characterization, and immune resistance mechanisms. Further, the growing arsenal of various immunotherapeutic agents, including novel classes of immune checkpoint inhibitors and oncolytic, chimeric antigen receptor T cell, and vaccine therapies, reinforces these efforts. This review will focus on the place of immunotherapy and future possible strategies in PDAC.

## Simple Summary

The development of immunotherapy for pancreatic adenocarcinoma has encountered numerous challenges, as immune checkpoint inhibitors are effective in only a small subset of patients. Key obstacles include tumor heterogeneity, the composition of tumor stroma, and the tumor microenvironment (TME). However, recent advancements in molecular characterization and our understanding of the TME have paved the way for innovative immunotherapy strategies. These advancements involve identifying predictive biomarkers, developing optimized treatment combinations, and targeting new mechanisms. Immunotherapy approaches should consider each patient’s individual characteristics to enhance effectiveness and address the resistance mechanisms seen with current therapies.

## 1. Introduction

Pancreatic ductal adenocarcinoma (PDAC) presents a significant challenge as a highly lethal malignancy, often diagnosed at an advanced stage, resulting in a 5-year overall survival (OS) rate of only 13% [1]. This places PDAC as the most fatal tumor, emphasizing the critical need for novel therapeutic approaches.

Standard treatments for advanced-stage PDAC include FOLFIRINOX (irinotecan, fluorouracil, leucovorin, and oxaliplatin) and NALIRIFOX (liposomal irinotecan, fluorouracil, leucovorin, and oxaliplatin) chemotherapies as well as gemcitabine/nab-paclitaxel(G/nP) combinations. Targeted therapy is an option for a small percentage (4 to 7%) of PDAC patients with specific genetic variants (*BRCA1/2, PALB2*, homologous recombination deficiency (*HRD*), *BRAF* V600E, Neurotrophic tyrosine receptor kinase (*NTRK*) mutation, and rearranged during transfection (*RET*) and neuregulin 1 *(NRG1)* gene fusion-positive tumors), as recommended by The National Comprehensive Cancer Network (NCCN) guideline [2].

**Figure 1:**
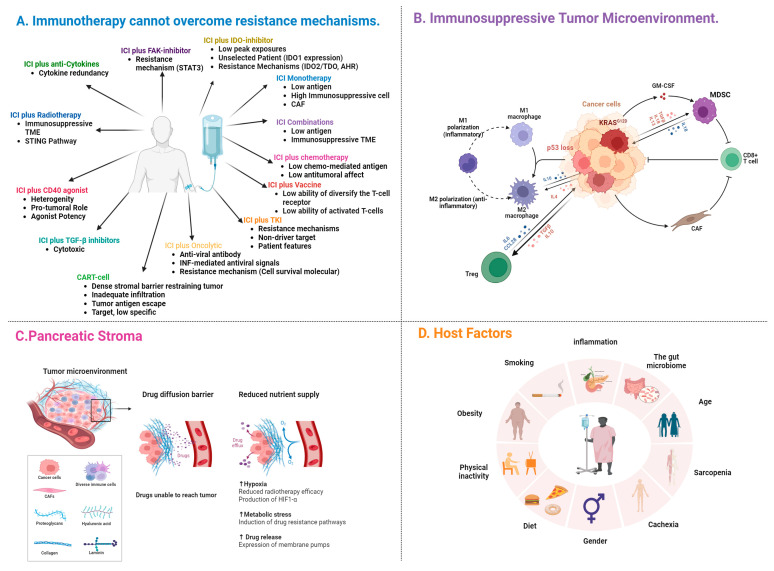
Reasons for the failure of immunotherapies in PDAC. (**A**) Reasons related to immunotherapeutic agents. (**B**) Reasons related to the tumor microenvironment. (**C**) Reasons related to pancreatic stroma. (**D**) Reasons related to host factors. Created in BioRender. Demir, T. (2025) https://BioRender.com/m54a934 (accessed on 7 February 2025).

Although immunotherapy has shown efficacy in various cancer types, its effectiveness in PDAC is limited except in a small subset of cases (1–2%) with specific genetic characteristics of microsatellite instability high (MSI-H). This limitation is attributed to factors such as the suboptimal impact of immunotherapeutic agents (Figure 1A), the tumor cells (TCs)’ low antigenicity, an immunosuppressive tumor microenvironment (TME) (Figure 1B), pancreatic stroma (Figure 1C), and host factors (Figure 1D) in PDAC [3].

Further, traditional immune predictive biomarkers have not reliably identified suitable candidates for treatment [4]. This review aims to comprehensively summarize the current understanding of immunology and immunotherapy treatments for PDAC, encompassing predictive biomarkers, resistance mechanisms, and ongoing immunotherapy studies, to facilitate the development of new treatment strategies for PDAC.

## 2. PDAC Immunology and Biomarkers

### 2.1. Immunology

The immune response in PDAC is relatively subdued compared with other solid tumors, primarily due to the abundance of immunosuppressive cells, unique TME traits, and molecular alterations [5]. Within the TME, immune cells are classified into lymphoid and myeloid clusters, each with distinct prognostic implications. In a study involving 104 patients with PDAC, the TME was analyzed, revealing that 51% of the patients were categorized as having a lymphoid immune profile, 22% as myeloid, and 37% as non-inflamed. Lymphoid clusters are associated with a favorable prognosis, while myeloid clusters are linked to a poorer prognosis [6]. Notably, the diverse immune cell (IC) populations within these clusters demonstrate varying prognostic significance. Certain ICs, including tumor-infiltrating T and B cells (TILs), N1 neutrophils, and type-1 dendritic cells (DCs), have been correlated with positive outcomes [7,8,9]. Conversely, regulatory T cells (Tregs), tumor-associated macrophages (TAMs), myeloid-derived suppressor cells (MDSCs), and cancer-associated fibroblasts (CAFs) are associated with unfavorable prognoses [9,10,11,12,13,14]. These disparities in IC functionality underline the complexities of devising effective immunotherapy strategies for PDAC.

The composition of the TME can also influence the survival outcomes of PDAC patients. Higher levels of neoantigens and CD8+ TILs within the TME are indicative of better prognoses [15,16]. However, even if the neoantigen level of the TME is high at diagnosis, it reduces over time, and myeloid cells replace lymphoid cluster cells [16]. These TME features might explain, in part, why immunotherapies do not work in treatment-refractory settings. Additionally, the inadequate timeframe for patients with advanced PDAC to establish a robust adaptive immune response may contribute to the failure of immunotherapies [3].

Furthermore, the intricate interplay between PDAC cancer cells and the pancreatic stroma significantly impacts the immune response. The stromal TME in the pancreas is poorly vascularized and has a dense extracellular matrix (ECM). This leads to hypoxia, which triggers the production of HIF1-α. HIF1-α then suppresses cytotoxic T cells through the programmed death-ligand 1 (PD-L1)/programmed cell death protein 1 (PD-1) pathway [17]. The immune response is also influenced by the physical characteristics and composition of the microenvironment, such as stiffness, pressure, and mechanical stress signaling [18]. The other stroma component, CAFs, have various features that can either promote or hinder tumor growth, depending on their interaction with the tumor and ICs. CAF protumor features encourage cancer cell growth and invasion, increase inflammatory cell levels, and reduce IC infiltration [3]. Also, the organization of collagen fibers can act as conduits for the guidance of ICs, highlighting the importance of ECM architecture [17]. So, this may affect the immune response, which is either pro-tumoral or antitumoral.

The coexistence of varying microenvironment compositions and the presence of KRAS mutations further compound the challenge of overcoming immunotherapy resistance in PDAC [19]. Notably, *KRAS* mutations have been linked to poor prognoses [20,21], and while the *KRAS G12C* mutation presents a potential treatment target, it is prevalent in only a small subset (1–2%) of PDAC patients [22]. However, 45%, 30%, and 13% of patients harbor *KRAS G12D, G12V*, and *G12R*, respectively [21].

In light of these intricacies, the development of efficacious immunotherapy strategies for advanced PDAC may necessitate the integration of immunotherapy with frontline PDAC chemotherapies, as opposed to pursuing immunotherapy as a standalone treatment [23].

### 2.2. Biomarkers

**Figure 2:**
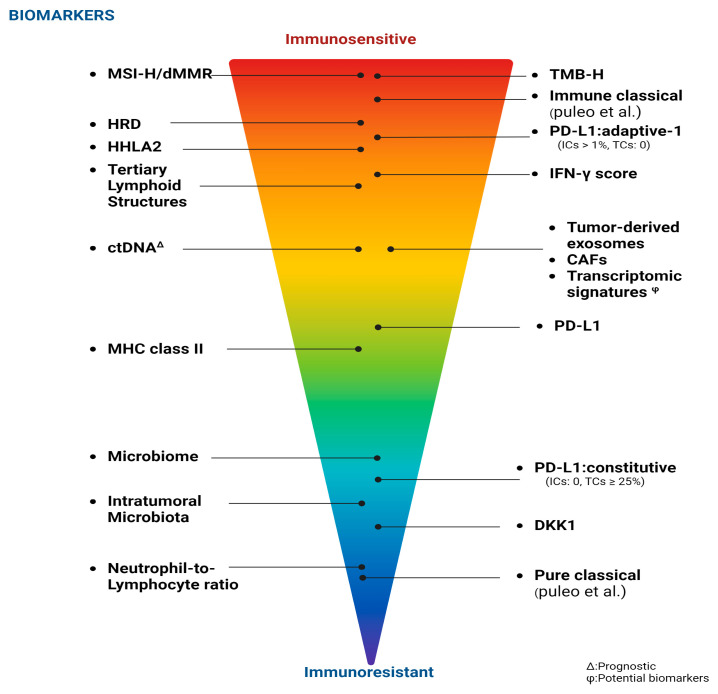
Biomarkers are systematically classified according to their sensitivity to immune responses [27]. Created in BioRender. Demir, T. (2025) https://BioRender.com/t48c033 (accessed on 7 February 2025).

Immunotherapy has demonstrated survival benefits in treating most solid tumors, yet its efficacy in patients with PDAC has been limited thus far. Nonetheless, certain PDAC patients have exhibited positive responses to immune checkpoint inhibitors (ICIs) [24]. Consequently, it is imperative to establish biomarkers for identifying such patients. Biomarkers originating from tumor tissues, such as PD-L1 expression, tissue-infiltrating lymphocytes, tumor mutational burden (TMB), and specific immune signatures, have displayed promise [25]. Moreover, various peripheral blood markers such as the neutrophil-to-lymphocyte ratio, circulating tumor DNA (ctDNA), and immune mediators like cytokines along with gut microbiota profiles are being assessed as predictive and prognostic markers in PDAC [4,26]. Nevertheless, as of yet, no immune-based biomarker has been validated for use in PDAC (Figure 2).

#### 2.2.1. TMB and MSI-H/Mismatch Repair Deficient (dMMR)

The NCCN guideline advocates for the use of ICIs in treating advanced PDAC patients displaying high MSI and TMB. Nevertheless, the potential benefits of high TMB as a predictive biomarker in PDAC remain uncertain, and the occurrence of MSI-H/dMMR and TMB-H in PDAC is relatively low, occurring in only 0.3% and 1.8% of cases, respectively. Furthermore, approximately 100% of PDAC cases exhibit a co-occurrence of high TMB and MSI-H/dMMR [28]. Additionally, high TMB is often linked to specific gene mutations (*ERBB2, POLE, BRCA2, ATR, ARID1A, KMT2D, SMARCA4*, and *BRAF*) [28,29,30]. While high TMB is characterized by having at least 10 mutations per megabase, there is no fixed cut-off for high TMB in PDAC. Recent studies emphasize the critical role of co-mutations in determining which patients would benefit most from ICIs [31]. Further research is warranted to accurately evaluate the role of TMB in PDAC treatment. Notably, MSI-H/dMMR stands as the singular proven predictor of ICI biomarkers for PDAC. A recent study underscored that MSI-H/dMMR PDACs displayed superior survival rates relative to MSI-stable (MSI-S)/proficient MMR (pMMR) counterparts prior to the initiation of immunotherapy [32]. Le et al. reported the effectiveness of pembrolizumab in MSI-H/dMMR solid tumors, including PDAC, which led to the inception of the KEYNOTE-158 study [33]. The study revealed the survival benefit of pembrolizumab in patients with MSI-H/dMMR non-colorectal solid tumors subsequent to the failure of standard therapy [34]. Additionally, a study showed that patients with MSI-H PDAC treated with ICIs derive durable benefits, irrespective of germline or somatic etiology [35]. In May 2017, the FDA, recognizing the efficacy of pembrolizumab, approved its use for treating MSI-H/dMMR solid tumors irrespective of the primary site, based on data from patients enrolled across five clinical trials [36].

#### 2.2.2. HRD

Individuals diagnosed with PDAC demonstrating *HRD*, particularly those with germline mutations in the *BRCA1* and *BRCA2* genes, have significantly improved when treated with platinum-based chemotherapy and PARP inhibitors [37]. For instance, olaparib, a recognized PARP inhibitor, has received FDA approval for first-line maintenance therapy in patients with metastatic PDAC who possess germline *BRCA* gene variants and maintain stability following a minimum of 16 weeks of chemotherapy [37]. Moreover, rucaparib is offered as a maintenance therapy option for patients with mPDAC carrying *BRCA1*, *BRCA2*, or *PALB2* variants, contingent upon the absence of disease progression after at least four months of platinum-based therapy [38]. Recent studies have further indicated that patients with metastatic, *HRD*-positive PDAC who undergo immunotherapy tend to experience prolonged survival outcomes [20,39,40]. Evidence also suggests that PARP inhibitors may elevate TMB and enhance immune responses within the TME, yielding additional benefits when utilized with ICIs. Notably, a recent clinical trial indicated that the combination of niraparib and ipilimumab for maintenance therapy successfully met the primary endpoint of progression-free survival (PFS). Conversely, combining niraparib and nivolumab resulted in less favorable PFS outcomes [40]. These findings emphasize the considerable predictive biomarker value of HRD in the context of PDAC immunotherapy.

#### 2.2.3. PD-L1

Extensive research has been conducted on the expression of PD-L1 in PDAC. It has been found that approximately 30–40% of PDAC cases exhibit PD-L1 expression, which has been associated with an unfavorable prognosis and low levels of CD8+ TIL [41,42]. PDAC is categorized into four distinct models based on the presence of PD-L1 on ICs and TCs: adaptive-1 (ICs > 1%, TCs: 0), adaptive-2 (ICs > 1%, TCs > 1% to < 25%), constitutive (ICs: 0, TCs ≥ 25%), and combined (ICs > 1%, TCs ≥ 25%) [43]. The adaptive-1 pattern is characterized by an inflamed TME with high levels of PD1+ T cells; CD3+, CD4+, and CD8+ lymphocytes; and a reduced number of CD68+ cells, including M2-polarized macrophages, and it has been linked to the best survival outcomes. Conversely, tumors with constitutive patterns are associated with poor clinical outcomes. The immune infiltration of CD68+ M2-polarized macrophages in PDAC plays a negative prognostic role [44]. Furthermore, increased infiltration of CD4+ and CD8+ cells is associated with a longer survival duration [45]. Despite being identified as a potent immunotherapy predictive biomarker in other solid tumors, the outcomes of PD-L1 expression are inconsistent in PDAC [42].

#### 2.2.4. H Long Terminal Repeat-Associating 2 (HHLA2)

HHLA2, a B7/CD28 family member, exhibits similarities to PD-L1 and plays a crucial role in regulating T-cell functions [46]. Comprehensive studies have demonstrated that HHLA2 is widely expressed in patients with a range of PD-L1-negative solid tumors [46,47]. Focusing on HHLA2 may offer a promising treatment option for patients resistant to PD-1/PD-L1 inhibitors [48]. Anti-PD-L1 therapy has demonstrated promising results in treating HHLA2 high solid tumors, including gallbladder cancer and melanoma [49,50,51]. Approximately 77% of PDAC patients express HHLA2, and its presence is significantly associated with a more favorable prognosis, underscoring its potential co-stimulatory role in this cancer type [52]. A recent study by Aydin et al. suggests that assessing HHLA2 expression in PD-L1-negative PDAC may provide valuable insights for predicting individual responses to immunotherapy [53]. This reinforces the significance of HHLA2 as a promising new target and predictive factor in the immunotherapy landscape.

#### 2.2.5. Interferon (IFN-γ)

IFN-γ plays a pivotal role in coordinating the immune response against tumors [54]. Recent studies have highlighted that IFN-γ is a crucial driver of PD-L1 expression in cancerous and healthy cells [55]. However, it is essential to note that the signaling pathway of IFN-γ can trigger a feedback loop that inhibits the immune response against tumors [56]. This loop activates the PD-1 signaling axis, which can lead to reduced effectiveness of T-cell cytotoxic responses. Tumors can manipulate this feedback mechanism to their advantage, further driving the progression of the cancer [57]. The IFN-γ score, a gene expression profile that includes genes such as IFNG, STAT1, CCR5, CXCL9, CXCL10, CXCL11, Indoleamine 2, 3-dioxygenase 1 (IDO1), PRF1, GZMA, and MHCII HLA-DRA, correlates with the expression of IFN-γ [58]. Moreover, the OpACIN-neo and DOMINI studies have revealed that a higher IFN-γ score is intricately linked to improved response rates to ICIs in patients with melanoma [59,60,61]. Furthermore, the data showed that high IFN-γ scores can potentially be utilized as predictive biomarkers to identify non-small-cell lung cancer patients who may also benefit from ICIs [62]. Numerous preclinical studies have demonstrated that IFN scores may be an important predictive factor for PDAC, particularly in the context of oncolytic therapies [63,64]. This finding deepens our understanding of the role of IFN-γ in cancer treatment and holds immense promise for the future of immunotherapy strategies in PDAC.

#### 2.2.6. Gut Microbiome

Recent studies have underscored the pivotal role of microbiome composition in predicting the efficacy of immunotherapy across various cancers, including PDAC [65,66]. Specific bacterial species, such as *ackermansia bacilli*, fecal bacilli, and *clostridium faecalis*, have been identified as effective enhancers of PD-1/PD-L1 antitumor immunotherapy [66]. Preclinical data demonstrate that the pancreases of patients with pancreatic cancer exhibit a higher prevalence of gram-negative proteobacteria, *Euryarchaeota*, and anaerobic *Synergistetes* compared with those of healthy individuals [67]. In addition, a multinational study has identified specific gut and oral microbial species associated with an increased risk of mortality related to PDAC [68]. These findings represent a promising opportunity for clinicians and researchers to develop more effective immunotherapy strategies for the treatment of PDAC. Numerous ongoing clinical trials are currently investigating the microbiome as a potential biomarker in this context (NCT05596370, NCT04922515, NCT05727020, NCT05523154, NCT05462496, NCT05580887, and NCT04638751).

#### 2.2.7. Intratumoral Microbiota

Furthermore, researchers have unveiled a significant novel constituent of the TME termed the intratumoral microbiota [69]. These bacteria primarily inhabit ICs and TCs and can impact the prognosis of PDAC patients by modulating ICs [67,70]. Additionally, the intratumoral microbiota contributes to the spatial heterogeneity of the TME by interacting with other cells, rendering it a crucial area necessitating further exploration [70]. Although the previous studies described how intratumoral fungi enhance *KRAS* mutations, mediating IL-33 secretion by PDAC, research on the association between the intratumoral microbiome and genomic mutations in PDAC is still inadequate [71]. Nevertheless, studies have shown that there are interactions between genomic alterations in PDAC and intratumoral microbes [72]. Further, a single-cell RNA-seq analysis of PDAC suggests itraconazole treatment inhibits the immunosuppressive aspects of the stroma and signals that promote tumor growth. Combining itraconazole with anti-PD1/Cytotoxic T-lymphocyte antigen 4 (CTLA-4) immunotherapy provides survival advantages in the PDAC model. Additionally, itraconazole has shown promise in hindering liver metastatic colonization in an mPDAC model [73]. While current research mainly focuses on intratumoral bacteria and fungi, more research must be done on the interactions between viruses and tumors. It has been established that the hepatitis B virus (HBV) and hepatitis C virus are associated with PDAC risk, and HBV expression can be detected in PDAC tissue [74,75].

#### 2.2.8. ctDNA

Liquid biopsy is an innovative diagnostic approach that analyzes circulating TCs or tumor-derived factors, particularly ctDNA. Highly sensitive liquid biopsy assays have been developed to detect and characterize minimal residual disease (MRD) [76]. MRD indicates the presence of TCs that have spread from the primary tumor to distant organs in patients who show no clinical or radiological signs of metastasis. After local treatment, it can also identify residual TCs, which may eventually lead to a local recurrence [77]. Importantly, detectable ctDNA is associated with poor prognosis of PDAC after definitive resection [78].

In addition, ctDNA has emerged as a valuable prognostic and predictive biomarker for a range of solid tumor types undergoing immunotherapy treatment [79]. It provides invaluable insights into the genomic evolution of tumors over time and helps us identify multiple metastases’ heterogeneity in patients [80]. Recent studies have indicated that ctDNA profiling may enable the earlier identification of relapse compared with imaging for PDAC patients [81,82]. Considerable opportunities exist to integrate ctDNA sequencing-based analyses into the routine clinical management of PDAC patients, encompassing mutation profiling for personalized treatment selection, molecular-based prognostication, and disease kinetics monitoring [83]. The CCTG PA.7 study recently assessed the efficacy of G/nP with or without durvalumab and tremelimumab for pancreatic PDAC, but unfortunately, the chemoimmunotherapy combination did not yield improvements in OS. As part of the clinical trial, exploratory baseline ctDNA sequencing prompted the acknowledgment that patients with *KRAS* wildtype tumors demonstrated markedly higher survival rates in the groups receiving combination immunotherapy and chemotherapy. These findings underscore the significant prognostic value of ctDNA-based *KRAS* mutation status in PDAC [20].

#### 2.2.9. Major Histocompatibility Complex (MHC) Class II

The expression of MHC class II is pivotal for antigen-presenting cells [84]. The role of MHC class II expression in TCs is still debatable. Studies showed that MHC-II expression in breast, colon, and larynx carcinoma correlates with a favorable prognosis [85,86,87]. Conversely, melanoma cells that express MHC-II molecules have been associated with a poor prognosis [88]. However, melanoma patients with MHC-II-positive tumors responded more favorably to ICIs [89]. LAG-3 is an immune checkpoint receptor that can be targeted through cancer immunotherapy approaches [90]. The functional role of MHC-II in PDAC still needs to be clarified. Recent findings suggest that the MHC-II/LAG-3 pathway contributed to CD4+ and CD8+ T-cell cytotoxicity toward MHC-II-positive PDAC cells [91]. The study also revealed that co-treatment with IFN-γ and/or MEK/histone deacetylase inhibitors induced tumoral MHC-II expression on MHC-II-negative, IFN-γ-resistant tumors [91], thereby presenting prospects for neo-antigen-based immunotherapy strategies.

#### 2.2.10. Dickkopf-1 (DKK1)

The *Wnt* signaling pathway is responsible for regulating a wide range of cellular processes crucial in embryonic development, including stem cell maintenance, cell fate decisions, and organ development [92]. DKK1 is a known antagonist of the canonical *Wnt/B-catenin* signaling pathway, but it has also been found to activate non-canonical *Wnt* signaling pathways and *PI3K/AKT* signaling [93]. Its expression in cancer varies across different types, with some instances showing overexpression and others showing under-expression [93,94]. In PDAC, high levels of DKK1 in patient serum are associated with lower OS rates, presenting a promising opportunity for early detection and monitoring of PDAC [95]. In cholangiocarcinoma and gastric cancer, inhibiting DKK1 in combination with immunotherapies and chemotherapies has shown promising results [96]. However, ongoing studies on DKK1′s role in immunotherapy for PDAC are still in the early stages [97].

#### 2.2.11. The Neutrophil-to-Lymphocyte Ratio (NLR)

The NLR, a significant factor in cancer prognosis, is linked to an unfavorable outcome in various cancer types, including PDAC, serving as a potential marker for the systemic manifestation of concurrent intratumoral inflammation and immune suppression [98]. Intratumoral neutrophils exhibit complex behavior as they secrete both immunosuppressive mediators and angiogenic factors in the TME, promoting tumor growth and metastasis. Additionally, circulating blood neutrophils directly interact with TCs, facilitating metastatic dissemination [99]. Neutrophil extracellular traps generated by CXLX5 and IL-17-activated tumor-associated neutrophils have been associated with diminished OS in PDAC, suggesting a pro-tumoral effect [100,101,102]. Furthermore, the inhibition of IL-17 can enhance sensitivity to ICIs in PDAC [102]. However, the plasticity and divergent functions of this cell population add a layer of complexity to our understanding of neutrophil behavior in PDAC, with distinct subtypes exhibiting contrasting effects on tumor progression [9].

#### 2.2.12. Other Potential Biomarkers

Puleo et al. have introduced a new classification system for PDAC based on tumor and stroma gene patterns, with potential implications for understanding and treating PDAC. This classification includes the pure basal-like, stroma-activated, desmoplastic, pure classical, and immune classical subtypes. The presence of a high number of immune infiltrates in the immune classical subtype suggests that it could be a target for immunotherapies. On the other hand, the pure classical subtype, which does not overexpress any immune checkpoint molecule, exhibits a lymphoid-enriched profile, indicating a potential avenue for non-ICIs [27]. This system holds the potential to guide therapy selection following validation.

In addition, tumor-derived exosomes, CAFs, and transcriptomic signatures have emerged as potential biomarkers for evaluating the efficacy of immunotherapy in PDAC [4,17,103]. These biomarkers can modulate the TME of PDAC [4]. Notably, several clinical trials are currently underway investigating the effectiveness of treatments targeting CAFs in combination with ICIs in patients with PDAC [3]. These trials hold promise and are expected to provide valuable insights into the role of these biomarkers in the context of immunotherapy for PDAC.

## 3. Resistance Mechanisms

The immune cycle is a series of steps that must operate accurately to stimulate an effective and clinically beneficial immune response against tumors. These steps include ensuring the immunogenicity of TCs, activating DCs efficiently, presenting antigens appropriately to T cells, and activating the T cells [104]. Each of these steps is crucial and contributes to the overall effectiveness of the immune response. However, it is essential to surmount the various immune resistance mechanisms before initiating the immune response. These mechanisms can hinder the immune cycle and must be understood and overcome for a successful immune response against PDAC. To date, immunotherapeutic strategies aimed at overcoming the mechanisms of immune resistance have demonstrated only modest efficacy in clinical trials (Table 1).

### 3.1. ICI Studies and Resistance Mechanisms

The studies on using monotherapy ICI for PDAC patients have not been successful, except in patients with MSI-H [34,105,106]. Various single-agent treatments like tremelimumab, ipilimumab, pembrolizumab, nivolumab, and durvalumab were tested on patients with mPDAC, with very limited efficacy observed [105,107,108,109,110,111]. Combination ICI treatments, while they showed some modest increase in tumor response, did not achieve a meaningful clinical efficacy endpoint. While subsets of PDAC patients positive for *HRD* were noted to have an improved objective response rate (ORR) of 42% with the combination of ipilimumab plus nivolumab, subsequent studies with dual checkpoint inhibitors reported only minimal to no responders in the overall unselected PDAC population [39,110,111]. The ineffectiveness of single-agent or combination ICIs is postulated to low tumor antigenicity, a high prevalence of immunosuppressive cells, and the restriction of immunocompetent cells by CAFs [15,110,111,112].

Chemotherapy agents have the potential to enhance tumor antigenicity and improve the activity of TILs, helping to overcome resistance to ICIs [113]. However, certain chemotherapy agents, such as 5-FU and irinotecan, may have immunosuppressive effects that could hinder the immune-mediated antitumor response [114]. Platinum-based agents and taxanes are preferred in clinical trials for PDAC, as they have been shown to induce immunogenic cell death and promote T-cell activation [115]. Regrettably, chemotherapy combinations with ICIs have not demonstrated favorable outcomes in subsequent treatment lines. For instance, a study revealed that combining gemcitabine and ipilimumab resulted in an ORR of only 14%, similar to the response observed with gemcitabine alone [116]. First-line treatments have had more encouraging results, such as the combination of G/nP with durvalumab and tremelimumab, which increased the DCR to 70.6%. However, this did not improve median OS compared with chemotherapy alone (9.8 months versus 8.8 months) [20]. In addition, early-phase trials integrating G/nP with pembrolizumab or nivolumab have yet to meet their primary endpoints. This deficiency is largely attributed to the fact that PDAC tumors release fewer antigens in response to chemotherapy than other solid tumors [117,118].

Given the immunosuppressive TME in PDAC, efforts to modulate the TME and enhance immunogenicity using the myeloid cell-activating receptors CD40 agonistic monoclonal antibodies have been integrated into the chemotherapy plus ICI regimen to amplify antigenicity and antitumor effects [119]. Initial findings from a phase Ib study indicate that sotigalimab, a CD40 agonistic monoclonal antibody, when combined with chemotherapy with or without nivolumab demonstrated good tolerability and clinical activity [120]. However, subsequent randomized phase II results revealed only modest improvements in OS. The sotigalimab/nivolumab/chemotherapy arm had a one-year OS of 41.3%, while the sotigalimab/chemotherapy arm had a one-year OS of 48.1%. In contrast, the nivolumab/chemotherapy arm met the primary endpoint with a one-year OS of 57.7% compared with historical controls, which had a one-year OS of 35% [121]. Given the benefit of the CD40 agonist with standard chemotherapy, a larger clinical study evaluating the CD40 agonistic IgG1 antibody mitazalimab, in combination with mFOLFIRINOX, is underway [122]. While we await the results of these clinical studies, evaluation is ongoing of the key mechanisms of resistance associated with the CD40 agonistic monoclonal antibody, including dendritic cell heterogeneity, the pro-tumoral role of regulatory B cells, and low agonist potency [13,123,124]. Further, Wattenberg et al. conducted a study using mouse models of PDAC, revealing that combined targeting of CD40 and dectin-1 can lead to enhanced antitumor immunity. They found that pancreatic tumors resistant to T cell-targeted immunotherapy could be eliminated with a systemic therapy of β-glucan and a CD40 agonist antibody. The effectiveness of this approach requires T cells but does not rely on traditional T-cell cytotoxicity or immune checkpoint pathways. Additionally, the antitumor response depends on IFN-γ and intratumoral macrophages. These results highlight the potential of targeting myeloid cell activation pathways in overcoming resistance to standard immunotherapy [125].

CXCR4 blockade has demonstrated enhanced TILs and synergistic effects with ICIs in PDAC mouse models [126]. A phase IIa clinical study evaluated the safety and efficacy of the CXCR4 antagonist BL-8040 (motixafortide) in combination with pembrolizumab and chemotherapy for patients with mPDAC. In cohort 1, 37 patients with chemotherapy-resistant disease received BL-8040 and pembrolizumab, resulting in a DCR of 34.5%, with 31% achieving SD and 3.4% achieving a PR. The mOS was 3.3 months overall and 7.5 months for second-line therapy recipients. Additionally, BL-8040 treatment increased CD8+ effector TILs and decreased MDSCs and Tregs. In cohort 2, 22 patients treated with BL-8040 and pembrolizumab alongside chemotherapy achieved an ORR of 32%, a DCR of 77%, and a median duration of response of 7.8 months [127]. These findings highlight the potential of combining CXCR4 and PD-1 blockade to enhance chemotherapy efficacy in PDAC, warranting further exploration through randomized trials. However, the redundancy of cytokines presents a challenge in this therapeutic approach [128].

In preclinical models, PARP inhibition and ICIs have demonstrated notable efficacy in tumors characterized by *HRD* [129,130,131]. Additionally, certain studies indicate that anti-CTLA-4 therapy may be more effective than anti-PD-1 therapy when administered alongside PARP inhibitors [129]. A randomized, phase Ib/II study investigated the efficacy of niraparib in combination with either nivolumab or ipilimumab for mPDAC patients who did not progress after ≥ 16 weeks of platinum-based therapy. The study consisted of two arms, niraparib plus nivolumab and niraparib plus ipilimumab, with the primary endpoints being safety and PFS rate at 6 months (PFS6) for each arm. The PFS6 for niraparib/nivolumab was 20.6%, while for niraparib/ipilimumab it was 59.6%. The maintenance therapy of niraparib/ipilimumab demonstrated superior PFS6, meeting the primary endpoint, whereas niraparib/nivolumab showed inferior PFS6. These findings underscore the potential of non-cytotoxic maintenance therapies in addressing the needs of patients with advanced PDAC [40].

IDO1 has emerged as a significant target for cancer therapy due to its critical role in tryptophan degradation [132]. The overexpression of IDO1 in TCs has been associated with poor survival outcomes in a variety of cancers, including PDAC [133]. Epacadostat, a potent IDO1 inhibitor, effectively obstructs the conversion of tryptophan in tumors and DCs [134]. The open-label phase I/II ECHO-203 study assessed the safety and efficacy of combining epacadostat with durvalumab in patients with advanced solid tumors, including PDAC. However, the ORR was only 12% [135]. The study was halted following the second interim analysis after the ECHO-301/KEYNOTE-252 study failed in melanoma patients [136]. Consequently, the effectiveness of IDO1 inhibition as a strategy to enhance the activity of anti-PD-1 therapy in cancer remains uncertain. Factors contributing to the failure of IDO1 inhibitors in combination with ICIs may include low peak drug exposures, the absence of patient selection based on IDO1 expression, compensatory expression of IDO2 or tryptophan 2,3-dioxygenase, and the activation of the immunosuppressive aryl hydrocarbon receptor [135,137].

Several multi-targeted kinase inhibitors (mTKIs) are currently undergoing clinical trials alongside immune checkpoint inhibitors (ICIs), while lenvatinib and axitinib have already become standard treatments when combined with immunotherapies [138,139]. Additionally, actionable driver mutations, such as those in the KRAS gene, may contribute to immune resistance [140]. This insight has prompted the initiation of new clinical trials to investigate the potential benefits of targeting these genetic alterations to enhance ICI efficacy. Research has indicated that the combination of specific agents such as trametinib (an MEK inhibitor) and ibrutinib (a BTK inhibitor) with chemotherapy has not yielded improved survival outcomes for patients with mPDAC [141,142]. A recent study evaluating selumetinib (another MEK inhibitor) in conjunction with durvalumab as a maintenance treatment did not meet its primary objective of improving PFS at four months [143]. Nonetheless, studies utilizing xenograft models suggest that targeting MEK, CDK4/6, BTK, and FAK may effectively slow tumor growth and enhance sensitivity to ICIs [144,145,146]. These findings suggest that the combination of ICIs and TKIs may represent a promising treatment strategy for pancreatic cancer. An interim analysis of the ALLEN regimen, which involves the combination of durvalumab, nab-paclitaxel, and lenvatinib, reported an ORR of 64.7% and a DCR of 94.1% in patients with mPDAC [147]. Furthermore, the combination of surufatinib (an mTKI), sintilimab (an anti-PD-1 antibody), and G/nP demonstrated encouraging results, with an ORR of 50.0% and a DCR of 91.7% in treatment-naive patients [148]. Additionally, the combination of surufatinib with camrelizumab, nab-paclitaxel, and S-1 displayed potential as a first-line treatment for mPDAC, achieving an ORR of 53.6% compared with 15.0% for the G/nP group [149]. While these trials are ongoing, the initial results indicate that combining mTKIs with immunotherapy may offer significant benefits for patients with mPDAC, and it warrants further investigation [148]. It is also essential to address the challenges posed by non-driver targets, downstream resistance mechanisms, and the activation of STAT3 signaling within this context [144,146].

In PDAC, extracellular adenosine (eADO) released from TCs suppresses immune responses. Quemliclustat (Q), a potent CD73 inhibitor, targets the production of eADO. Elevated CD73 expression is seen in 40–60% of PDAC cases and is linked to poor outcomes. The ARC-8 study examined the safety and efficacy of Q, alone or combined with zimberelimab (anti-PD-1), alongside G/nP in treatment-naive mPDAC patients. Results showed that adding Q was safe and well tolerated without increasing chemotherapy toxicity. The median follow-up for survival was 21 months, with an mPFS of 8.8 months. These results support further development of Q for mPDAC treatment [150].

Radiotherapy (RT) affects the TME, damaging blood vessels and causing inflammation. This inhibits immune cell infiltration and promotes the accumulation of radioresistant suppressor cells, leading to increased tumor hypoxia and weakened anti-cancer effects. However, irradiation can also stimulate an immune response through immunogenic cell death, resulting in an “in situ” vaccination effect [151]. The combination of ICI with RT has been studied in the neoadjuvant setting for PDAC, with one study reporting an ORR of 60% and a remarkable R0 resection rate of 90%. Additionally, the 12-month PFS and OS rates were reported as 64% and 72%, respectively, indicating promising antitumor activity [152]. A recent study examined the effects of combining the GVAX vaccine, a-PD-1, and stereotactic body radiation (SBRT) after chemotherapy for locally advanced PDAC. This combination enhanced the antitumor immune responses and increased immunosuppressive M2-like tumor-associated macrophages (TAMs). Incorporating SBRT reduced the distance between PD-1+CD8+ T cells and TCs, improving survival rates and guiding future radioimmunotherapy studies targeting M2-like TAMs [153]. Further, in an early-phase clinical trial, combining GVAX, cyclophosphamide, pembrolizumab, and the *CSF1R* inhibitor IMC-CS4 demonstrated an increase in activated CD8+ T cells [154]. Resistance mechanisms related to this combination include increased infiltration of Tregs, MDSCs, and the activation of the STING pathway [155]. A recent study suggests that the *ATM* inhibitor AZD1390 may help overcome these resistance mechanisms when combined with radiation, enhancing the CD8+ T cell-mediated adaptive immune response and sensitizing pancreatic cancer to immunotherapy [156].

### 3.2. Oncolytics and Resistance Mechanisms

The efficacy of oncolytic viruses (OVs) lies in their capacity to elicit an immune response by releasing tumor antigens and eliminating cancer cells. This phenomenon has the potential to transform “cold” tumors, like PDAC, into “hot” tumors, thereby enhancing the efficacy of immunotherapies in combination treatments [157]. Notably, global approval has been granted to four OVs and one non-oncolytic virus for cancer treatment, including *H101* (adenovirus) for nasopharyngeal carcinoma, *T-VEC* (herpes simplex virus 1 (HSV1)) and *ECHO-7* (echovirus) for melanoma, *teserpaturev* (HSV1) for glioblastoma, and *nadofaregene firadenovec* (adenovirus) for bladder carcinoma [158].

Several types of OVs, such as adenovirus, HSV, reovirus, and parvovirus, have undergone early-phase clinical trials involving PDAC patients [159]. Among these, reovirus and adenovirus have been the focus of extensive studies [160].

#### 3.2.1. Reovirus

Reolysin is a non-genetically modified reovirus administered intravenously that selectively targets TCs, including those activated by *RAS* mutations, and enhances their responsiveness to ICIs [161,162].

A phase II study involving chemotherapy-naive patients diagnosed with mPDAC assessed the efficacy of combining reolysin with gemcitabine, with the primary objective of evaluating the DCR. Among the 34 patients evaluated, results indicated modest efficacy; one patient achieved a PR, while 23 patients exhibited SD. Pharmacodynamic analyses revealed reovirus replication within the PDAC tumors, which was associated with apoptosis and an upregulation of PD-L1 expression [163]. In a phase Ib study, researchers investigated the addition of reolysin to pembrolizumab alongside a single chemotherapy agent—either gemcitabine, irinotecan, or 5-fluorouracil—in an unselected PDAC patient population during second-line treatment. This study included 11 patients and reported a DCR of 27%. Notably, one patient maintained a PR for 17.4 months, and two additional patients demonstrated SD for 9 and 4 months, respectively. Furthermore, the study observed reoviral replication in tumor biopsies collected during treatment, an increase in intratumoral CD8+ T cells, and elevated levels of caspase-3 along with the expression of the innate immune response genes PD-L1 and IDO1. Changes in peripheral T-cell clonality were noted, particularly among patients who experienced clinical benefits, highlighting shifts in immune gene expression [164]. Another recent study examined the efficacy of reolysin in combination with pembrolizumab as a second-line therapeutic option for advanced PDAC patients. This study involved 12 patients and demonstrated a DCR of 42%, with 1 patient achieving a PR and 4 patients experiencing SD. Importantly, this combination therapy did not result in an increase in immune-related toxicity. A thorough analysis of tumor biopsies taken during treatment revealed substantial immunomodulatory effects, including viral replication, the infiltration of CD8+ T cells and NK cells interacting with PD-L1+ cells, and a reduction in peripheral Tregs, among patients who benefited from the therapy [165]. These findings contributed to the design of the phase I/II GOBLET study, which evaluates the combination of reolysin with atezolizumab and G/nP for first-line treatment in PDAC patients. Preliminary data from this study indicate a favorable ORR of 62% among PDAC patients, along with a 6-month PFS rate of 72.9% and a 6-month OS rate of 82.1%. The ORR reported is more than twice the average ORR observed in historical control trials of G/nP, which is approximately 25% [166]. The evidence thus suggests that reolysin engenders synergistic effects when combined with ICIs by effectively reversing the immunosuppressive tumor microenvironment. A new cohort has been added to the ongoing GOBLET study to further evaluate the effectiveness of reolysin in combination with mFOLFIRINOX, with or without atezolizumab, as the first-line treatment for patients with mPDAC. (Eudra-CT: 2020-003996-16) [167].

#### 3.2.2. Adenovirus

Adenoviruses have been employed in the treatment of patients with PDAC through both intratumoral and intravenous administration in combination with chemotherapy during early-phase clinical trials. Specific adenoviruses utilized include Ad5-yCD/mutTK (SR39), Ad5-yCD/mutTKSR39, LOAd703, ONYX-015, and VCN-01 [168]. In one particular study, 14 patients received intravenous VCN-01 in combination with a treatment regimen known as G/nP. This approach was determined to be both feasible and safe, demonstrating promising results, with an ORR of 50% among mPDAC patients [169]. Presently, a phase IIb open-label randomized clinical trial, titled VIRAGE, is underway to evaluate the efficacy of G/nP with or without VCN-01 in patients diagnosed with mPDAC (NCT05673811) [170]. Furthermore, another clinical trial involved 21 patients who were administered intratumoral injections of LOAd703, also in combination with G/nP [171]. This trial reported an ORR of 44% and a DCR of 94%. The outcomes from these trials have facilitated the continuation of clinical research exploring adenovirus-based oncolytic therapies alongside RT, chemotherapy, and ICIs. A completed non-randomized phase I trial administered intratumoral injections of the herpes simplex virus T-VEC to 30 patients with unresectable PDAC; however, the results from this trial are not yet available (NCT00402025). Additionally, in a phase II study involving patients with mPDAC, parvovirus H-1PV was introduced both intravenously and intratumorally under ultrasound guidance. This study enrolled seven patients, reporting a PFS of 2.5 months and an OS of 6 months [172].

#### 3.2.3. Challenges with OV Therapy

OVs are widely regarded as less toxic than conventional cancer treatments. Furthermore, OVs are considered a more cost-effective and sustainable option for immunotherapy compared with adoptive therapy or chimeric antigen receptor T-cell (CAR-T) therapy. Nevertheless, challenges persist in the implementation of OV therapy, despite notable advancements in both preclinical and clinical trials [173]. The principal challenges associated with oncolytic therapy include ensuring both logistical and biological safety. This encompasses appropriate storage at −80 °C, preparation in a sterile biosafety cabinet, as well as the careful handling and administration of varying doses and the timing for both initial and subsequent injections, particularly in the case of intratumoral injections for accessible tumors [174]. Each oncolytic virus carries specific challenges and advantages. For instance, adenoviruses, including Oncorine, Onyx-015, Delta-24-RGD, and enadenotucirev, encounter difficulties related to their tropism for various tissues and limited viral spread. Similarly, HSV-1 viruses, such as T-VEC, OH2, and G47D, present their own challenges, including the risk of latent infection and significant pathogenicity. There have been instances reported of healthcare workers contracting infections while handling T-VEC, and a study noted that 8.4% of household contacts exhibited symptoms of cold sores [159]. Additionally, reolysin presents challenges related to gene editing. However, adenoviruses offer several advantages, such as the capacity to generate high viral concentrations, accessibility, the potential for genetic manipulation, robust lytic activity, and the ability to enhance immunomodulatory agents in combination therapy [175]. HSV-1′s large genome allows for genetic modification and ensures replication occurs solely within infected cells. Reoviruses can be administered intravenously at elevated doses without inducing significant toxicity, demonstrating antitumoral activity while enhancing the effects of immunomodulatory agents in combination therapy, leading to the upregulation of PD-L1 [159,175].

#### 3.2.4. Resistance Mechanisms Associated with OV Therapy

The resistance mechanisms associated with ICI plus OV treatments encompass several factors, including antiviral antibodies, IFN-mediated antiviral signals, and cell survival molecular mechanisms that protect TCs against viral infection and infection-induced oncolysis [169]. A promising strategy to address these resistance mechanisms may involve the concurrent administration of both intratumoral and intravenous OVs alongside other therapeutic agents.

### 3.3. Vaccines and Resistance Mechanisms

Cancer vaccine therapies aim to create an immune response against specific tumor-related antigens. These vaccines include peptide, DNA, whole TC, vector-based, mRNA, and DC vaccines. Despite many clinical trials of cancer vaccines for PDAC, there are still significant challenges in achieving good results [176]. Two main factors limiting effectiveness are the lack of diversity in the T-cell receptor repertoire and the low number of activated T cells infiltrating tumors [177].

#### 3.3.1. Dendritic Cell (DC) Vaccines

Dendritic cells come from a patient’s blood and are enriched with peptides, DNA, RNA, or tumor materials. When used as a vaccine, these enhanced DCs move to the lymph nodes to activate T cells. Sipuleucel-T, the first DC-based vaccine approved by the FDA in 2010, treats prostate adenocarcinoma. A trial with 42 patients who had advanced PDAC tested the effectiveness of a combination of MUC1-cytotoxic T cells and MUC1-mRNA-transfected DCs (MUC1-DCs) along with gemcitabine. The results showed an mOS of 13.9 months and a 1-year OS rate of 51.1%. One patient experienced a CR, three had a PR, and 22 showed stable disease. Importantly, there were no severe adverse events from the vaccine. Additionally, patients who received more than 1 × 10^7^ MUC1-DCs per injection had a longer median survival time than those who received fewer (16.1 months vs. 3.2 months; *p* = 0.0036), indicating that the dosage may affect results [178]. Another study looked at combining Wilms tumor gene 1 (*WT1*) peptide-pulsed DC vaccination with gemcitabine (DCGEM) for PDAC patients who are HLA-A*2402 positive. This treatment combination was well tolerated and showed potential for triggering antitumor T-cell responses, making it a promising option for treating PDAC, especially in patients without liver metastases [179]. Additionally, a recent double-blinded clinical trial in Japan tested a DC vaccine using *WT1* peptides (TLP0-001) for patients with PDAC that did not respond to chemotherapy [180]. A different study tested allogeneic tumor lysate-loaded autologous monocyte-derived DCs in patients who had resection of their PDAC and no signs of recurrence after standard treatment. This study included 10 patients, all of whom had no serious vaccine-related adverse events. Every patient showed an immune response to the vaccine, indicated by increased numbers of Ki67+ and activated PD-1+ circulating T cells. They also displayed T-cell reactions to their own TCs. At a follow-up after 25 months, seven out of ten patients showed no signs of disease recurrence or progression [181].

#### 3.3.2. Peptide Vaccine

The peptide vaccine GV1001 is designed to target the human telomerase reverse transcriptase catalytic subunit (h*TERT*) class II 16mer [182]. A phase II clinical trial involving patients diagnosed with advanced PDAC demonstrated that 63.2% of participants exhibited an immune response to the vaccine. The mOS for immune responders was 216 days in contrast to 88 days for non-responders [183]. However, subsequent findings from a randomized phase III study indicated that the addition of the GV1001 vaccine to gemcitabine and capecitabine did not yield a survival benefit for patients with mPDAC [184]. Survivin, a member of the inhibitor of apoptosis protein family, is expressed across various malignancies, rendering it a compelling target for therapeutic interventions. A phase II trial evaluated the survivin 2B peptide (SVN-2B) vaccine in patients with PDAC. The results were encouraging, as the combination of SVN-2B with IFNβ peptide vaccination therapy demonstrated both safety and feasibility, suggesting that extended peptide vaccination regimens may offer benefits to individuals with PDAC. Furthermore, the study emphasized the potential of type 1 IFN as a promising adjuvant for peptide vaccination therapy [185].

#### 3.3.3. Listeria Monocytogenes (LM)-Based Vaccine

LM-based vaccine vectors have shown the ability to effectively target and activate DCs in vivo, utilizing the immunogenic properties of infectious vectors to stimulate both adaptive and innate immune responses. Following comprehensive safety evaluations, the vaccine was administered in conjunction with ICI [186]. Nonetheless, this combination did not achieve its primary endpoints during a randomized phase II study, with the mOS recorded at 5.9 months and the ORR at merely 4% in the treatment group. Importantly, alterations in the immune microenvironment, including an increase in CD8+ T cells and a decrease in CD68+ myeloid cells, were noted exclusively among long-term survivors in the combination arm [187].

#### 3.3.4. Neoepitope Vaccine

OSE2101 is a neoepitope vaccine comprising 10 synthetic peptides that specifically target CEA, HER2-Neu, p53, MAGE2, and MAGE3—biomarkers frequently overexpressed in PDAC. The vaccine was developed with HLA-A2-restricted presentation in mind. A clinical study was conducted to evaluate the safety and efficacy of OSE2101, both as a standalone treatment and in combination with the anti-PD1 agent nivolumab, in patients with metastatic PDAC following FOLFIRINOX induction chemotherapy. The primary objective of this study was to assess OS at 12 months. The findings indicated that OSE2101 administered alone exhibited a favorable safety profile and was associated with an extended time to treatment failure. However, the combination therapy with nivolumab was linked to inferior outcomes, prompting the discontinuation of that treatment arm. Following recommendations from the independent data monitoring committee, the study will proceed with a revised design that compares maintenance therapy with FOLFIRI alone versus FOLFIRI in combination with OSE2101 (NCT03806309) [188].

#### 3.3.5. mRNA Vaccine

Recent advancements in mRNA vaccine technology have facilitated the development of personalized neoantigen vaccines for cancer treatment [189]. These vaccines effectively activate immune cells and allow for efficient delivery during clinical trials [190]. In a phase I clinical trial initiated by Rojas et al., patients with surgically resectable PDAC were administered a combination of adjuvant atezolizumab, autogene cevumeran (an individualized mRNA neoantigen vaccine), and mFOLFIRINOX. The primary objectives of the trial included evaluating the immune response induced by the vaccine, assessing 18-month RFS, and determining overall feasibility. After a median follow-up of 18 months, it was observed that patients with vaccine-expanded T cells (designated as responders) demonstrated a longer median RFS, which was not reached, compared with non-responders, who exhibited a median RFS of 13.4 months (*p* = 0.003). This combination therapy resulted in significant T-cell activity, which may potentially delay the recurrence of PDAC [191].

### 3.4. CAR-T Cell Treatment and Resistance Mechanisms

CAR-T cells represent an innovative approach to cancer treatment. These modified cells are designed to recognize cancer cell surface markers independently of HLA [192]. While CAR-T cells show promise across various cancer types, their application to solid tumors presents specific challenges, including limited effectiveness, high toxicity, and logistical complexities [192,193]. Additionally, issues such as inadequate tumor infiltration due to dense surrounding tissue [18] and tumor antigen escape are recognized as significant resistance mechanisms in PDAC [113].

A study by Beatty and colleagues evaluated the safety and effectiveness of mesothelin-specific CAR-T cell therapy (CAR-T-meso-cells) in six patients diagnosed with advanced PDAC. The patients received CAR-T-meso-cells through intravenous infusion three times a week over three weeks. The results indicated no severe adverse effects, with two patients achieving disease stabilization and displaying PFS durations of 3.8 and 5.4 months. Imaging assessments revealed stable metabolic activity in three patients, while one exhibited a significant 69.2% reduction in metabolic activity [194].

Additionally, another trial investigated CAR-T cell therapy targeting HER2 in patients with PDAC. Following pre-treatment conditioning, patients received CAR-T-HER2 cells, resulting in PFS durations of 5.3 and 8.3 months, with SD being the most favorable outcome [195].

In a further phase Ib study, researchers examined autologous CAR-T cells targeting CLDN18.2 in patients with PDAC. Two of the six patients assessed demonstrated SD, while three experienced PD following an initial response [196].

### 3.5. Resistance Mechanisms: Patients’ Characteristics

Patient characteristics play a significant role in shaping PDAC immunology and influencing the responses to immunotherapy. Gender is an essential factor, as heightened immune activity is often observed in women, which can be attributed to the effects of 17-β-estradiol and its associated receptors [197,198]. Additionally, obesity, a well-known risk factor for developing PDAC, presents a complex challenge due to its impact on immunotherapy sensitivity, altering the immune system and microbiota and thereby leading to a poor immunogenic response [199,200]. Dietary factors like a high-fat diet can create a pro-tumoral inflammatory microenvironment by activating oncogenic pathways, such as *KRAS* and *COX2* [201]. Further, chronic pancreatitis and cachexia contribute to pancreatic inflammation and foster a suppressive TME [202,203]. Conversely, lifestyle factors—including regular physical activity and balanced nutrition—can positively influence gut microbiota and overall immune responses [204,205]. Furthermore, aspects such as smoking status and sarcopenia may impact tumor immunogenicity, underscoring the importance of lifestyle interventions in PDAC management [206,207].

The liver serves as the primary site of metastasis in PDAC, affecting over 60% of patients, followed by the lungs and peritoneum in approximately 30% of cases [208]. The liver’s unique immune environment limits the effectiveness of CD8+ T cells, whereas lung metastases are characterized by increased immune cell infiltration [209,210]. Consequently, liver metastasis represents a significant mechanism of immune resistance in PDAC, presenting challenges for effective treatment strategies.

## 4. Future Strategies

Numerous clinical trials are currently being conducted to evaluate the efficacy of combination immunotherapy in patients diagnosed with PDAC, as detailed in Table 2. The findings from these ongoing studies will inform future strategies that emphasize the use of combination therapies and the optimization of host characteristics. These methodologies frequently incorporate innovative drugs that target novel pathways.

### 4.1. Novel Immunotherapy Approaches

Novel ICIs, including anti-TIGIT, LAG-3, VISTA, and CTLA-4, present promising new strategies for combination therapies in PDAC [49,211,212,213]. Research utilizing single-cell data indicates that chemotherapy significantly alters the TME in PDAC, potentially contributing to resistance against immunotherapy by diminishing the interactions between TIGIT on CD8+ T cells and its receptor on cancer cells. This information supports the consideration of anti-TIGIT immunotherapies in subsequent treatment lines following chemotherapy progression in PDAC [49]. Further, a preclinical study has demonstrated encouraging results when combining ICIs with LAG-3 antagonists in murine models of PDAC, which led to a favorable transformation of the TME [212]. Furthermore, in a preclinical study, botensilimab, an Fc-enhanced anti-CTLA-4 antibody, exhibited effectiveness comparable to or superior to conventional chemotherapeutics, such as gemcitabine, nab-paclitaxel, and cisplatin, controlling 7 of 10 tumor cells in combination with chemotherapy [213].

Allogeneic lysate DC vaccination presents a promising strategy for addressing resistance mechanisms in PDAC. A phase I study assessed the safety and immunogenicity of allogeneic tumor-derived DCs in a cohort of ten patients who had undergone surgical resection for PDAC. Positive responses to the vaccine were observed in all participants, as evidenced by increased frequencies of Ki67+ and activated PD-1+ circulating T cells. Additionally, T-cell reactivity against the patients’ tumors was demonstrated. At the most recent follow-up, with a median duration of 25 months (ranging from 15 to 32 months), seven patients exhibited no signs of disease recurrence or progression [181]. Moreover, advancements in next-generation vaccines, particularly neoantigen vaccines, have shown considerable promise when used with ICIs to treat solid tumors [214]. Noteworthy data emerged from a phase I trial involving 16 patients with resected PDAC that investigated a personalized neoantigen vaccine utilizing mRNA–lipoplex nanoparticles derived from each patient’s tumor tissue. Participants received atezolizumab, the personalized cerumen-based neoantigen vaccine, and a chemotherapy regimen (mFOLFIRINOX) to evaluate their effects on immune response, 18-month RFS, and overall feasibility. Notably, eight patients who received the vaccine demonstrated prolonged RFS [191]. However, further validation through larger randomized clinical trials is essential to directly compare these findings with the current standard of care and establish the therapeutic benefits for patients with PDAC.

Numerous strategies are being developed to enhance CAR-T cell therapy for treating solid tumors [215,216]. These strategies include introducing therapeutic transgenes such as cytokines and chemokines, utilizing OVs, and employing bispecific engagers and checkpoint antibodies. Additionally, direct targeting of the CAR antigen at the tumor site aims to augment the efficacy of CAR-T cells [217,218,219,220,221,222]. A preclinical study demonstrated that loading a virus onto CAR-T cells in vitro, followed by the activation of the native TCR in vivo, significantly improved therapeutic outcomes compared with using OVs or CAR-T cells in isolation. The observed enhanced efficacy was attributed to an increased presence of the virus in the lymph nodes and tumors, alterations in memory cell differentiation profiles, and improved functionality of the CAR-T cells. Furthermore, this combined approach facilitated greater epitope spreading within endogenous T-cell populations, highlighting the potential of integrating CAR-T cell therapy with OV therapy through systemic delivery. This methodology allows for TCR activation without requiring lymphodepletion in murine models, necessitating further investigation [223]. Moreover, other cellular immunotherapies, including CAR-NK cells and CAR-TILs, present promising avenues as potential strategies to address tumor heterogeneity and resistance mechanisms in PDAC [224,225].

### 4.2. New Targetable Immune Pathways

Multiple studies have established that the inhibition of mitogen-activated extracellular signal-regulated kinase (MEK) leads to the activation of the signal transducer and activator of the transcription 3 (STAT3) signaling pathway, which is associated with therapeutic resistance and the ongoing proliferation of PDAC cells. Notably, the concurrent inhibition of Janus kinases (JAKs) and STAT3 (referred to as STAT3i) in combination with MEK has demonstrated the ability to overcome this resistance, owing to the activation of parallel feedback mechanisms [226]. Furthermore, the combined inhibition of MEK and STAT3 has exhibited beneficial effects on tumor fibrosis. This approach enhances the infiltration of CD8+ T cells while diminishing the presence of immunosuppressive Tregs and MDSCs within the TME. As a result, these changes contribute to a reduction in tumor burden and improved survival outcomes in genetically engineered mouse models of PDAC. It is important to emphasize that the observed antitumor effects are dependent on T-cell activity. However, this alteration in the TME is accompanied by the sustained expression of immune checkpoint proteins, including PD-L1/PD-1 and CTLA-4 [227]. Therefore, the combination of MEK and STAT3 inhibition with ICIs presents a promising therapeutic strategy. Current clinical trials are underway to evaluate the safety and efficacy of this combination in patients with metastatic PDAC [228].

The innate immune system is critical for safeguarding the body against pathogens and cancer cells, and it can convert “cold” tumors into “hot” tumors that are more amenable to therapeutic intervention [229]. A significant component of this system is the Cyclic GMP-AMP Synthase (cGAS)–Stimulator of IFN Genes (STING) pathway [229]. The cGAS enzyme recognizes DNA and activates the type-1 IFN pathway, which is essential for effective antitumor immunity [229,230]. The combination of ICIs with STING agonists presents a promising strategy for treating solid tumors, including PDAC [230]. Furthermore, emerging approaches in innate immunotherapy include the application of anti-CD47 antibodies, IL-17, and anti-SIRPα [102,231].

Glycogen synthase kinase-3β (GSK-3β) is a serine/threonine kinase that is currently being studied as a potential target for cancer therapies due to its important role in various critical cellular processes [232,233]. Research has shown that GSK-3β plays a significant role in pathways related to MHC-I antigen processing and the antigen-processing machinery, particularly the signaling pathways mediated by IFN-γ and the NF-κB pathway [234,235,236]. Further, preclinical studies have demonstrated that inhibiting GSK-3β with 9-ING-41 (elraglusib) enhances efficacy of TILs and CD8+ T cells to tumor cells [237,238]. Furthermore, our recent clinical trial indicates that 9-ING-41 (elraglusib) has a favorable toxicity profile with G/nP for PDAC [239]. The potential synergy of combining chemotherapy and immunotherapy with 9-ING-41 (elraglusib) in the management of PDAC warrants further investigation.

Recent preclinical studies have provided substantial support for the combination of TGF-β inhibitors and ICI, yielding consistently positive preclinical results across diverse tumor types. However, as these combinations progress to clinical trials, the incorporation of TGF-β inhibitors frequently demonstrates no meaningful benefit beyond that afforded by the current generation of ICIs alone [240]. Notably, approximately 60% of PDAC cases present with an *SMAD4* mutation [241], leading to the loss of its tumor suppressor function in canonical TGF-β signaling [242]. Consequently, this loss of *SMAD4* results in reduced T-cell recruitment and concomitant suppression of the immune response [243]. Therefore, the targeted modulation of the *SMAD4*/TGF-β signaling pathway represents a promising therapeutic strategy, mainly when combined with other agents for treating PDAC [244].

TME modulation has shown promising strategies for overcoming immune resistance mechanisms in PDAC. This strategy is currently under investigation with several drugs, including those inhibiting nicotinamide adenine dinucleotide phosphate (NADPH) oxidase 4 (NOX4), Pin1, fibroblast activation protein (NCT03932565), HPN536, and BTK-inhibitor [141,245,246,247,248].

PDAC presents significant challenges for effective systemic therapy delivery due to the dense fibrotic stroma surrounding the tumor [249]. Hyaluronan, a glycosaminoglycan in the ECM of the TME, is linked to aggressive metastatic disease, drug resistance, and poor prognosis [250]. Pegvorhyaluronidase alfa (PEGPH20) is a PEGylated recombinant human hyaluronidase aimed at remodeling the stroma to improve systemic agent distribution. A phase III trial showed that combining pegvorhyaluronidase alfa with G/nP increased the ORR in patients with high hyaluronan levels but did not improve survival [251]. Additionally, CEND-1, a cyclic peptide targeting αV integrins and neuropilin-1, enhances drug delivery to tumor sites. Recent studies indicate that CEND-1 combined with G/nP has a favorable safety profile and promising activity in metastatic PDAC patients [252]. A proton-driven transformable nano-vaccine offers the potential to address the challenges posed by the dense fibrotic TME. This vaccine induces a strong immune response with minimal toxicity. In acidic endosomes, it transforms to deliver antigenic peptides into the cytoplasm, enhancing tumor immunity. Mouse model studies demonstrate its effectiveness in tumor growth inhibition, and in combination with anti-PD-L1 antibodies, it leads to improved survival and tumor regression [253]. Overall, these therapies show promise for combination immunotherapy strategies for PDAC.

### 4.3. Optimization of Patient Characteristics

Modification of host factors can also improve PDAC’s sensitivity to immunotherapies. The microbiota’s interaction with ICIs makes it a potential target for therapeutic intervention [254]. Modifying the microbiota can be achieved through antibiotics or fecal transplants (NCT03785210). Identifying specific bacteria and pathways that modulate the immune response could lead to targeted interventions. In an early clinical trial, adding Salmonella-IL2 to FOLFIRINOX for mPDAC resulted in nearly doubling median survival [255]. Additionally, the combined effects of exercise and nutrition can enhance the efficacy of immunotherapies [205,256]. More research on the modification of host factors is needed.

In response to all this information, we have summarized the potential combination treatments in Table 3.

## 5. Conclusions

The current application of immunotherapies as systemic treatments for PDAC has yet to be effectively tailored to the unique characteristics of this disease. Although prior clinical trials involving various immunotherapies, including ICIs, oncolytic therapies, vaccines, and CAR-T cell therapies, have yielded disappointing results, the insights garnered from these studies remain invaluable. Researchers have successfully identified new potential therapeutic targets and specific aspects of the TME in PDAC; however, it is critical to acknowledge that existing immunotherapies have not resulted in favorable patient outcomes. Consequently, developing novel immunotherapy options, whether utilized as monotherapy or combined with other agents such as chemotherapy, must be grounded in a robust biological rationale. Given the rapid disease progression associated with PDAC, there is a pressing need for innovative clinical trial designs. The meticulous selection of patients through predictive biomarkers is essential in this process. Furthermore, addressing resistance mechanisms is crucial for enhancing the effectiveness of immunotherapy strategies. Ultimately, the successful treatment of PDAC necessitates a multimodal approach that targets the TCs, the microenvironment, and host factors, thereby fostering immunogenicity within the tumors.

## Figures and Tables

**Table 1:** Completed immunotherapy studies in **advanced** pancreatic adenocarcinoma.

Completed Immunotherapy Studies in Advanced PDAC						
Drug	Clinical Trial Identifier	Sample Size (*n*)	ORR (%)	mPFS (Months)	mOS (Months)	Grade ≥ 3 AEs (%)
ICI Studies						
ICI-Monotherapy						
Tremelimumab	NCT02527434	20	0	N/A	4	30
Ipilimumab	NCT00112580	27	0	N/A	N/A	11
Pembrolizumab	NCT01295827	1	0	N/A	N/A	0
Durvalumab	NCT02558894	33	0	1.5	3.6	6
Nivolumab	NCT01928394	18	0	1.4	5.1	0
ICI Combination Therapy						
Durvalumab plus tremelimumab	NCT02558894	32	3.1	1.5	3.1	22
Ipilimumab plus nivolumab	NCT01928394	21	0	1.4	4	0
ICI plus Chemotherapy						
G/nP plus durvalumab plus tremelimumab	NCT02879318	119	30.3	5.5	9.8	84
G/nP plus pembrolizumab	NCT02331251	17	N/A	9.1	15	53
G/nP plus nivolumab	NCT02309177	50	18	5.5	9.9	96
Gemcitabine plus ipilimumab	NCT01473940	21	14	2.7	6.9	48
ICI plus CD40 monoclonal antibody						
Chemotherapy plus nivolumab plus sotigalimab	NCT03214250	35	31	6.7	10.1	29
Mitazalimab plus mFOLFIRINOX	NCT04888312	70	40	7.7	14.3	80
ICI plus CXCR4 antagonist						
Motixafortide plus pembrolizumab	NCT02826486	37	3.4	2.7	3.3	18.9
Motixafortide plus pembrolizumab plus chemotherapy	NCT02826486	22	32	7.8	N/A	40.9
ICI plus PARP inhibitor						
Niraparib plus nivolumab	NCT03404960	46	7.1	1.9	13.2	22
Niraparib plus ipilimumab	NCT03404960	45	15.4	8.1	17.3	50
ICI plus IDO-1 inhibitor						
Epacadostat plus durvalumab	NCT02318277	15	0	1.9	N/A	14.7
ICI plus MEK inhibitor						
Selumetinib plus durvalumab (maintenance)	NCT04348045	70	N/A	3.7	10.1	23.6
Cobimetinib plus ipilimumab plus nivolumab	NCT01928394	30	10	3	6.2	0
ICI plus mTKI						
Lenvatinib plus nab-paclitaxel plus Durvalumab	NCT05327582	17	64.7	7.8	8.1	0
Surufatinib plus cemrelizumab plus chemotherapy	NCT05218889	28	53.6	9.17	15.57	N/A
Surufatinib plus sintilimab plus G/nP	NCT05481476	15	50	N/R	N/A	33
ICI plus CD73 inhibitor						
Quemliclustat plus G/nP plus zimberelimab	NCT04104672	93	38	5.4	13.9	85
Oncolytic Studies						
Adenovirus						
EUS-injected adenovirus (Ad5-yCD/mutTK(SR39)rep-ADP (Ad5-DS) plus gemcitabine	NCT02894944	9	11	11.4	N/A	0
VCN-01 plus G/nP	NCT02045602	14	50	6.7	13.5	28.5
Ad5-yCD/mutTKSR39rep-hIL12 plus chemotherapy	NCT03281382	12	N/A	N/A	18.6	5.8
Intratumoral LOAd703 injections plus G/nP	NCT02705196	21	44	N/A	8.7	4.7
HSV						
T-VEC	NCT00402025	17	N/A	N/A	N/A	N/A
Parvovirus						
H-1PV (IV and US-guide IT)	NCT02653313	7	28.5	2.5	6	N/A
Reovirus						
Reolysin plus pembrolizumab plus chemotherapy	NCT02620423	11	9.1	2	3.1	18.2
Reolysin plus gemcitabine	NCT00998322	34	2.9	3.4	10.2	27
Reolysin plus carboplatin plus paclitaxel	NCT01280058	36	19	4.9	7.3	56
Reolysin plus pembrolizumab	NCT03723915	12	8.3	1.9	6.3	15
Reolysin plus atezolizumab plus G/nP	Eudra-CT: 2020-003996-16	13	62	N/A	N/A	N/A
Vaccine Studies						
Dendritic Cell Vaccine						
MUC1-mRNA transfected DCs and T cells plus gemcitabine	N/A	42	9.5	N/A	13.9	31
Wilms tumor gene 1 peptide-pulsed DC plus gemcitabine	UMIN-000004855	10	0	N/A	8	Negative DLT, protocol was terminated.
Autologous DC pulsed with allogeneic tumor cell lysate (adjuvant)	N/A	10	N/A	N/R	N/R	10
Peptide Vaccine						
Gemcitabine plus capecitabine plus telomerase peptide vaccine GV1001	N/A	354	N/A	6.58	8.36	22
Survivin 2B peptide vaccination plus IFNβ	UMIN-000012146	36	0	N/A	3.3	41.7
Cyclophosphamide/GVAX plus Listeria-mesothelin (CRS-207) plus nivolumab	NCT02243371	51	4	2.2	5.9	35.3
OSE2101 vaccine alone (Arm A) or in combination with nivolumab (Arm B) or FOLFIRI (Arm C) after induction with FOLFIRINOX (maintenance)	NCT03806309	Arm A (10) Arm B (10) Arm C (9)	Arm A (10) Arm B (0) Arm C (11)	Arm A (2.7) Arm B (2.2) Arm C (6.3)	Arm A (9.6) Arm B (7.9) Arm C (11.5)	Arm A (0) Arm B (20) Arm C (10)
RNA Neoantigen Vaccine						
Atezolizumab plus autogene cevumeran plus mFOLFIRINOX (adjuvant)	NCT04161755	34	N/A	N/R	N/R	6
CAR-T cell Studies						
Mesothelin-CAR-T cell	N/A	6	16	2 pts: 3.8 and 5.4	N/A	0 (non-hematologic)
HER2-CAR-T cell	NCT01935843	11 (2 PDAC)	0	5.3 and 8.3	N/A	0 (non-hematologic)
Claudin 18.2-CAR-T cell	NCT04404595	11 (6 PDAC)	0	N/A	N/A	0 (non-hematologic)

**Table 2:** Ongoing immunotherapy clinical trials in pancreas adenocarcinoma.

Ongoing Immunotherapy Clinical Trials in Pancreas Adenocarcinoma					
Immunotherapy	Phase	Clinical Trial Identifier	Experimental Arm	Control Arm	Primary Outcome
Immune Check Inhibitor					
ICI plus chemotherapy (neoadjuvant)	II	NCT06094140	mFOLFIRINOX plus durvalumab	None	Feasibility
ICI plus chemotherapy	I/II	NCT06454448	Adebrelimab plus decitabine plus G/nP	None	DLT, ORR
ICI plus chemotherapy plus anti-CD39	II	NCT06119217	Budigalimab plus G/nP plus TTX-030	G/nP	PFS
ICI plus chemotherapy plus anti-CD73 (neoadjuvant)	II	NCT04940286	Durvalumab plus G/nP plus oleclumab	None	MPR, safety
ICI (bispecific) plus chemotherapy	II	NCT06153368	Cadonilimab+ plus mFOLFIRINOX	None	ORR
ICI plus RT	II	NCT04361162	Nivolumab plus ipilimumab plus radiation	None	ORR
ICI plus ablation	I	NCT06378047	Pembrolizumab plus irreversible electroporation	None	Safety
ICI plus ablation	II	NCT03080974	Nivolumab plus irreversible electroporation	None	Safety
ICI plus chemotherapy plus RT(PLUSAR)	II	NCT06359275	Toripalimab plus G/nP plus RT	None	PFS
ICI plus PARP inhibitor	II	NCT05093231	Pembrolizumab plus olaparib in mPDAC with TMB-high	None	ORR
ICI plus PARP inhibitor	II	NCT04548752	Pembrolizumab plus olaparib in mPDAC with BRCA1/2 mutation	Olaparib	PFS
ICI plus anti-TIGIT plus chemotherapy	I/II	NCT03193190	Atezolizumab plus tiragolumab plus G/nP	G/nP	ORR, safety
ICI plus CD40 agonist plus chemotherapy	I/II	NCT03193190	Atezolizumab plus selicrelumab plus G/nP	G/nP	ORR, safety
ICI plus anti-TIGIT plus CD40 agonist	I/II	NCT05419479	Zimberelimab plus domvanalimab plus APX005M (maintenance)	FOLFIRI	Safety, PFS
ICI plus anti-VEGF	I/II	NCT05000294	Atezolizumab plus tivozanib	None	ORR
ICI plus anti-VEGF	II	NCT03074513	Atezolizumab plus bevacizumab	None	ORR
ICI plus anti-VEGF plus chemotherapy	I/II	NCT03193190	Atezolizumab plus bevacizumab plus G/nP	G/nP	ORR, safety
ICI plus anti-adenosine receptor plus chemotherapy	I/II	NCT03193190	Atezolizumab plus AB928 plus G/nP	G/nP	ORR, safety
ICI plus MEK inhibitor	I/II	NCT03193190	Atezolizumab plus cobimetinib	None	ORR, safety
ICI plus MEK inhibitor plus STAT3 inhibitor	I	NCT05440942	Trametinib plus ruxolitinib plus retifanlimab	None	MTD, safety
ICI plus mTKI (maintenance)	II	NCT04887805	Lenvatinib plus pembrolizumab	None	PFS
ICI plus PEGylated recombinant human hyaluronidase	I/II	NCT03193190	Atezolizumab plus PEGPH20	None	ORR, safety
ICI plus anti-IL6R plus chemotherapy	I/II	NCT03193190	Atezolizumab plus tocilizumab plus G/nP	G/nP	ORR, safety
ICI plus bispecific IL-2v	I/II	NCT03193190	Atezolizumab plus RO6874281	None	ORR, safety
ICI plus CXCR4 antagonist	I/II	NCT03193190	Atezolizumab plus BL-8040	None	ORR, safety
ICI plus CXCR4 antagonist plus G/nP	II	NCT04543071	Cemiplimab plus BL-8040 plus G/nP	None	ORR
ICI plus STING agonist	I/II	NCT05070247	Pembrolizumab plus TAK-500	None	ORR, safety
ICI plus anti-semaphorin 4D	I/II	NCT05102721	Avelumab plus pepinemab	None	Safety
ICI plus vaccine plus oncolytic (adjuvant)	I	NCT05846516	ATP150/ATP152 plus VSV-GP154 plus ezabenlimab	None	DLT, DFS
ICI plus ADC plus chemotherapy	I	NCT03816358	Anetumab ravtansine with nivolumab, nivolumab and ipilimumab, or gemcitabine and nivolumab	None	MTD
ICI plus TLR-3	I/II	NCT05927142	Durvalumab plus rintatolimod	None	Safety, ORR
ICI plus TLR-7 plus RT	II	NCT03915678	Atezolizumab + BDB001 + RT	None	Safety, ORR
ICI plus TIL	II	NCT01174121	Pembrolizumab plus TIL	None	ORR
ICI plus anti-CD73	I	NCT05431270	Tislelizumab plus PT199	None	MTD, DLT
ICI plus rIL-2	I/II	NCT05086692	Pembrolizumab plus MDNA11	None	Safety
ICI plus FGFR-inhibitor plus chemotherapy	II	NCT05945823	Pembrolizumab plus futibatinib plus mFOLFIRINOX	None	ORR
Oncolytic					
Adenovirus plus RT	II	NCT04739046	Ad5-yCD/mutTKSR39rep-ADP plus RT	None	ORR
Adenovirus plus chemotherapy	II	NCT05673811	VCN-01 plus G/nP	G/nP	OS and Safety
Adenovirus plus CART cell	I	NCT05057715	VCN-01 plus huCART-meso cells	None	Safety, DLT
Adenovirus plus ICI	I	NCT05076760	Intratumoral MEM-288 plus nivolumab	None	MTD, Safety, ORR
Adenovirus plus chemotherapy plus ICI	I/II	NCT02705196	IT-LOAd703 plus G/nP plus atezolizumab	None	DLT
HSV	I	NCT03086642	T-VEC	None	Safety
HSV plus chemotherapy	I	NCT03252808	HF10 plus G/nP	None	Safety
HSV	I	NCT01935453	OrienX010	None	Safety
Reovirus	I/II	Eudra-CT: 2020-003996-16	Reolysin plus atezolizumab plus G/nP or mFOLFIRINOX	None	ORR, Safety
TME Modulator					
Anti-CD40 plus chemotherapy	Ib/II	NCT04888312	Mitazalimab plus mFOLFIRINOX	None	DLT, ORR
Anti-CD40 plus ablation	I	NCT06205849	Intratumoral mitazalimab plus irreversible electroporation	None	Safety
Trispecific T-cell activator	I	NCT03872206	HPN536	None	ORR, safety
Microbiome					
ICI plus microbiome modulation	I	NCT05462496	Pembrolizumab plus antibiotics (neoadjuvant)	None	Immune response
ICI plus microbiome modulation	II	NCT03785210	Nivolumab plus tadalafil plus oral vancomycin	None	BOR
Vaccine					
Vaccine plus chemotherapy (maintenance)	II	NCT03806309	OSE2101 plus FOLFIRI	FOLFIRI	OS
Vaccine plus chemotherapy	I	NCT04157127	Th-1 DC vaccine plus SOC (adjuvant)	None	MTD
Vaccine plus ICI plus IDO1 inhibitor	II	NCT03006302	CRS-207 plus pembrolizumab plus epacadostat +/− cyclophosphamide and GVAX pancreas vaccine	None	MTD
Vaccine plus ICI plus chemotherapy	II	NCT06498518	IMM-101 plus pembrolizumab plus gemcitabine	None	ORR
Vaccine plus ICI plus RT	II	NCT05721846	Nivolumab plus ipilimumab plus TGFβ-15 peptide vaccine plus radiotherapy	None	Safety
Vaccine plus ICI plus vasodilator	II	NCT05014776	CRS-207 plus pembrolizumab plus ipilimumab plus tadalafil	None	ORR
Cell Treatment					
CAR-T cell	I	NCT02349724	Anti-CEA CAR-T cell	None	Safety
CAR-T cell	I/II	NCT02587689	Anti-MUC1 CAR-T cells	None	Safety
CAR-T cell	I	NCT02706782	Meso-CAR-T cells	None	Safety
CAR-T cell	I	NCT03323944	huCAR-T-meso CAR-T cells	None	Safety
CAR-T cell	I	NCT06196294	GPC3/Meso-CAR-γδT	None	Safety
CAR-T cell	I/II	NCT02830724	Anti-hCD70 CAR-T cells	None	Safety
CAR-T cell CRISPR-Cas9-Engineered	I/II	NCT05795595	CTX131	None	Safety, ORR
CAR-T cell plus ICI	I/II	NCT03182803	Meso-CAR-T cells plus anti-CTLA-4/PD-1	None	Safety
CAR-T cell plus ICI	I/II	NCT03030001	Meso-CAR-T cells plus anti-PD-1	None	Safety
TIL CRISPR-Cas9-Engineered	I/II	NCT04426669	Tumor infiltrating lymphocytes	None	MTD, safety
Autologous T cells	I/II	NCT05194735	Neoantigen specific TCR-T cell	None	MTD, DLT, ORR, safety

**Table 3:** Potential future strategies.

Future Strategies	
Nowel Drugs	Potential Combination Strategies
MEK inhibitors STAT3 inhibitors	Combination with ICIs and other immunotherapies (OVs, vaccines)
Anti-TIGIT Anti-LAG-3 Anti-VISTA New generation anti-CTLA-4	Combination with ICIs and chemotherapy
New generation vaccines (neoantigen, allogenic lysate-DC)	Combination with ICIs and chemotherapy
CAR-T cells CAR-NK cells CAR-TILs	Combination with OVs, ICIs, cytokines, chemokines
Anti-GSK-3β	Combination with ICIs and other immunotherapies (OVs, vaccines) and chemotherapy
STING agonist Anti-CD47 antibodies IL-17 Anti-SIRPα	Combination with ICIs
Anti-TGF-β	Combination with ICIs
TME modulation (anti-NOX4, anti-Pin1, anti-fibroblast activation protein, anti-HPN536 and BTK-inhibitor)	Combination with ICIs
Pegvorhyaluronidase alfa (PEGPH20) CEND-1	Combination with ICIs and chemotherapy
Modification of host factors (microbiota, Salmonella-IL2)	Combination with ICIs and chemotherapy

## Abbreviations

ADC	Antibody–drug conjugate
AEs	Adverse events
AHR	Aryl hydrocarbon receptor
BTK	Bruton tyrosine kinase
CAF	Cancer-associated fibroblast
CAR-NK	Chimeric antigen receptor natural killer cell
CAR-T	Chimeric antigen receptor T cell
CAR-TILs	Chimeric antigen receptor tumor-infiltrating lymphocyte cells
CD	Cluster of differentiation
CEA	Carcinoembryonic antigen
ctDNA	Circulating tumor DNA
CXCR4	C-X-C chemokine receptor type 4
DC	Dendritic cell
DFS	Disease-free survival
DLT	Dose-limiting toxicity
ECM	Extracellular matrix
FAK	Focal adhesion kinase
FGFR	Fibroblast growth factor receptor
GM-CSF	Granulocyte–macrophage colony-stimulating factor
GSK-3β	Glycogen synthase kinase-3β
HER2	Human epidermal growth factor receptor 2
HHLA2	H long terminal repeat-associating 2
HRD	Homologous recombination deficiency
HSV	Herpes simplex virus
ICI	Immune checkpoint inhibitor
IDO-1	Indoleamine 2,3-dioxygenase
IFN-γ	Interferon
IL	Interleukin
LAG3	Lymphocyte activation gene 3
MDSC	Myeloid-derived suppressor cell
MEK	Mitogen-activated extracellular signal-regulated kinase
MHC	Major histocompatibility complex
mOS	Median overall survival
mPFS	Median progression-free survival
MPR	Major pathological response
MSI-H/dMMR	Microsatellite instability high/mismatch repair deficient
MTD	Minimal toxic dose
N/A	Not available
NK	Natural killer
NOX4	Nicotinamide adenine dinucleotide phosphate (NADPH) oxidase 4
ORR	Objective response rate
OVs	Oncolytic viruses
PARP	Poly(ADP-ribose) polymerase
PD1	Programmed death 1
PD-L1	Programmed death-ligand 1
RT	Radiotherapy
STAT	Signal transducer and activator of transcription
STING	Stimulator of INF genes
TCR	T-cell receptor
TDO	Tryptophan-2,3-dioxygenase
TGF-β	Transforming growth factor beta
TIGIT	T-cell immunoreceptor with immunoglobulin and ITIM domain
TIL	Tumor-infiltrating lymphocyte
TLR	Toll-like receptor
TMB-H	Tumor mutation burden high
TME	Tumor microenvironment
Treg	Regulatory T cells
VEGF	Vascular endothelial growth factor
VISTA	V-domain Ig suppressor of T-cell activation
